# Effect of Zinc Supplementation on Urate Pathway Enzymes in Spermatozoa and Seminal Plasma of Iraqi Asthenozoospermic Patients: A Randomized Controlled Trial

**DOI:** 10.22074/ijfs.2020.5760

**Published:** 2019-11-11

**Authors:** Abdul Razzaq S. Alsalman, Lamia A. Almashhedy, Abdulsamie H. Alta'ee, Mahmoud H. Hadwan

**Affiliations:** 1 College of Medicine, University of Babylon, Hilla, Iraq; 2 Chemistry Department, College of Science, University of Babylon, Hilla, Iraq; 3 College of Pharmacy, University of Babylon, Hilla, Iraq

**Keywords:** Adenosine Deaminase, 5'-Nucleotidase, Uric Acid, Xanthine Oxidase, Zinc Supplementation

## Abstract

**Background:**

Uric acid (UA) is crucial for sperm metabolism as it protects seminal plasma against oxidative dam- age. Zinc also plays a central role in sperm metabolism. The current study was designed to investigate the role of zinc supplementation on qualitative and quantitative properties of seminal fluid, in parallel with the UA level and urate pathway enzymes in the semen of patients with asthenozoospermia.

**Materials and Methods:**

The study was designed as a randomized controlled trial of 60 asthenozoospermic subfertile men. The current study, which was conducted during one year, involved 60 fertile and 60 asthenozoospermic subfertile men belonging to Hilla City, Iraq. Semen samples were obtained from the participants before and after treatment with zinc supplements. The levels of UA, xanthine oxidase (XO), adenosine deaminase (ADA) and 5'-nucleotidase (5'-NU) activities were determined in spermatozoa and seminal plasma of both groups.

**Results:**

UA levels (P=0.034) and 5'-NU activity (P=0.046) were significantly lower but ADA (P=0.05) and XO (P=0.015) activities were significantly higher in infertile men than in healthy men. Treatment with zinc sulfate induced an increase in UA (P=0.001) level and 5'-NU activity (P=0.001), but a decrease in ADA (P=0.016) and XO (P=0.05) activities.

**Conclusion:**

Zinc supplementation restores UA levels and the activities of enzymes involved in the urate pathway (XO and ADA) in the seminal plasma and spermatozoa of patients with asthenozoospermia, to reference values. Sup- plementation of Zn compounds enhances the qualitative and quantitative properties of semen (Registration number: NCT03361618).

## Introduction

Male infertility as an underlying cause of subfertility, is observed in approximately 20% infertile couples. Although the percentage reaches up to 40% couples, both female and male factors are accounted. Thus, half of all infertility cases are caused by male-related factors (1). Asthenozoospermia, or low sperm motility, may be caused by sperm structural or functional deficiencies, a harmful effect of seminal plasma, or a combination of these factors. There are numerous factors, such as oxidative stress and nutritional insufficiency, contributing to male infertility (2). Although zinc is found in most food types, the World Health Organization (WHO) estimates that 33% of the world population suffer from zinc deficiency (3). Zinc is a fundamental micronutrient essential for different biochemical functions in mammals. Zinc has two forms: the first is found in the muscles, most of which is inadequately exchangeable and closely bound to high molecular weight ligands, such as nucleic acids, nucleoproteins, and metalloproteins; and the second form is freely exchangeable and is tightly bound to citrate and amino acid (4). Zinc is involved in cell differentiation and proliferation by regulating protein synthesis, nucleic acid metabolism, and secretion of growth hormone, testosterone, prolactin, and other steroid hormones. Zinc acts as a structural component of several enzymes that participate in DNA synthesis and transcription. It is also attached to zinc-binding proteins of more than thousands of transcription factors where these factors supply a platform for interaction with proteins or nucleic acids (5).

The level of production of reactive oxygen species (ROS) in male reproductive tract, is of crucial importance because of the possible noxious properties of high concentrations of ROS; these noxious effects affect the physical properties of semen quality (6). Normal levels of ROS are essential for the regulation of normal sperm functions (7), motility, hyperactivation, and capacitation and acrosome reaction and sperm-oocyte fusion (8). Conversely, elevated concentrations of ROS can negatively affect semen quality. Pathological effects of ROS include increased lipid peroxidation (LPO) levels, decreased sperm motility, DNA damage, and apoptosis (9). Oxidative stress-induced sperm damage has been explained to be a significant contributing factor in 30-80% of all cases of male subfertility (10). Men with subfertility who produce high concentrations of ROS, are seven times less likely to create a pregnancy compared with those producing low concentrations of ROS. ROS production can be aggravated by environmental, infectious, and lifestyle etiologies (11).

Uric acid (UA) is the final compound of nucleotide catabolism. It reacts with oxidants as an essential watersoluble antioxidant. Consequently, UA has a possible function in resisting spermatozoal oxidative damage. The regulatory effector (adenosine) and cell energy compound, adenosine triphosphate (ATP), were maintained by adenine nucleotide catabolism that embodies central pathways of the intermediary metabolism. In most tissues, an ideal adenine nucleotide pool is provided via a specialized mechanism that correlates with adenosine 5’ monophosphate (AMP) metabolism (12). Two fundamental enzyme sequences normally participate in the catalysis of the original AMP metabolism pathway. The first is AMP deaminase, which catalyzes the deamination of AMP to produce inosine monophosphate (IMP). The second is 5’-nucleotidase (5’-NU), which catalyzes the dephosphorylation of AMP to produce adenosine. The catabolism process further includes the conversion of adenosine to inosine via adenosine deaminase (ADA) catalytic activity. Xanthine oxidase (XO) catalyzes the terminal degradation of purine bases that generate UA, which is the final product of purines catabolism. ROS are produced during the enzymatic reaction of XO (13).

The proposed defensive role of UA against ROS in human seminal plasma has not been adequately tested. Only few research studies investigating the levels of UA in seminal fluid and the antioxidative resistance function of UA (14), have been published in scientific journals.

Furthermore, considerable controversies and inconsistencies exist in the literature. Although UA is an essential part of the total antioxidant status of human seminal fluid, which is reduced in subfertile subjects (12), another study (14) documented that UA levels were reduced in patients with normozoospermia. Consequently, the accurate UA level in seminal fluid is still undetermined.

Although few reports have investigated the association between subfertility and UA levels in semen, to the best of our knowledge, no study has reported the effects of asthenozoospermia treatments, such as oral zinc supplementation, on the activity of urate-related enzymes, which are important in fertility of the humans. The present study was designed to investigate the effect of zinc treatment on the qualitative and quantitative properties of semen, as well as UA concentrations and urate-related enzymes in the seminal fluid of men with asthenozoospermia.

## Materials and Methods

### Objectives

The primary objective was to determine the effect of zinc treatment on the qualitative and quantitative properties of semen. The secondary objective was to investigate the effect of zinc treatment on the UA concentrations and urate-related enzymes in the seminal fluid of men with asthenozoospermia.

### Study design

The trial was designed as a randomized clinical trial. The randomized trial was designed as a parallel group, superiority trial with 1:1 allocation ratio. No placebo was used in the current study. Double blind trial was applied for both the investigator and subject. The time of random allocation was completed immediately after the assessment for eligibility. Sixty male partners (age 32.8 ± 3.57 years) with subfertility were included in the present study. All couples were consulted at the Infertility Clinic of the Babylon Teaching Hospital of Maternity in Babylon governorate, Hilla City, Iraq from July 2011 to July 2012. Sample size was estimated according to Kadam and Bhalerao (15) method.

n=[2(ZaZ1-β)2σ2]Δ2

n=number of samples Zα, is 1.96 (constant that obtained from ref (15)). Z1-β, is 0.8416 (constant that obtained from ref (15), when a study has 80% power.

σ = population standard deviation, it is equal to 1.23 according to published article. Δ= the difference in effect of two interventions. i.e., it is equal to 3.66-3.04= 0.62.

**Fig 1:**
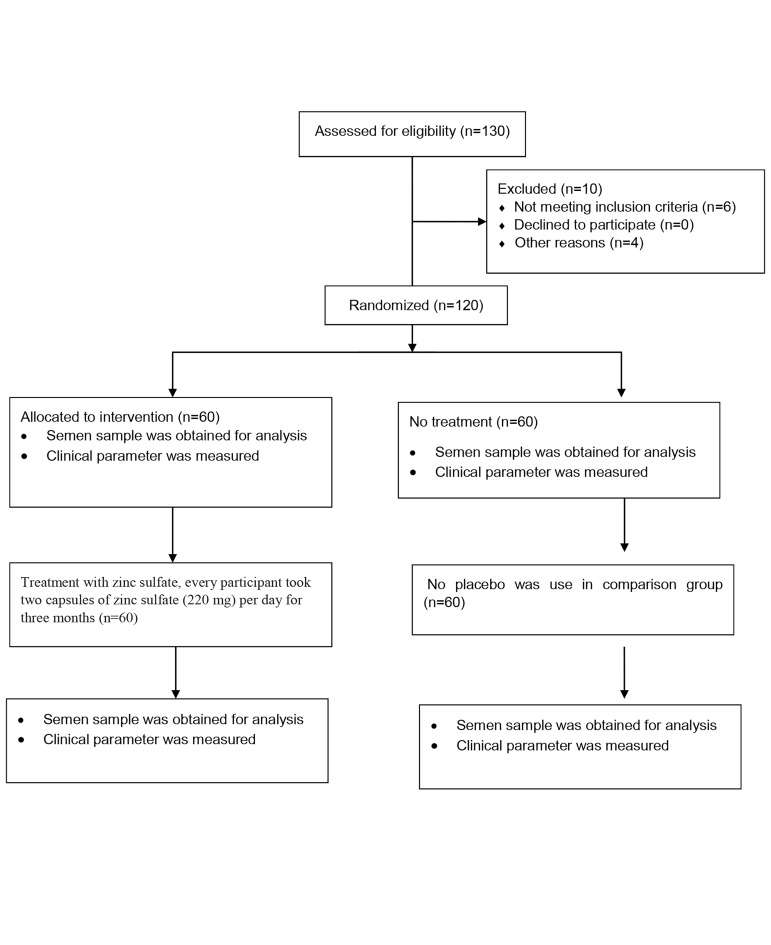
Flow of participants recruitment in the present trial.

The method that was used to generate the random allocation sequence, includes using a random-numbers table. Type of randomization is block randomization works by randomizing participants within blocks and allocates an equal number to each group. Professor Abdul Razzaq Alsalman generated the random allocation sequence, enrolled participants, and assigned participants to interventions. Figure 1 shows the flow of participants recruitment in this trial. A physical examination was completed, and complete medical history was recorded for each participant. Subjects who were administered with antioxidant supplementation or any other medication during the study period, were excluded from the study. The study was approved by The Institutional Research Ethics Committee [Ethics Committee (University of Babylon/College of Science), Reference number of approval: 545], and informed consent was obtained from all individual participants included in the study. The criteria for inclusion in the study were presence of asthenozoospermia, absence of varicocele, female factor infertility, and endocrinopathy. Smokers were excluded from the study because of their distinguished low antioxidant concentrations and elevated seminal ROS concentrations. The selection criteria for inclusion into the fertile group, were as follows: those with children born within the previous year, absence of asthenozoospermia, endocrinopathy, and varicocele. All seminal analyses were performed based on the 2010 WHO recommendation. These analyses included checking for semen pH, sperm motility, semen volume, semen concentration, normal sperm, and round cell morphology (16). No changes were made to the methods during the study period.

### Preparation of spermatozoa and seminal plasma for biochemical analysis

Spermatozoa were separated from the seminal plasma 1 hour after semen collection. Subsequently, 2 ml of seminal fluid was centrifuged at 1600 g for 15 minutes at 2°C and stored at -30°C until biochemical assessment. The pellet was washed with ten volumes of NTPC medium (a medium composed of NaCl, NaH_2_PO4_4_, Na_2_HPO_4_, Tris, EDTA, CaCl_2_, and D-glucose) and centrifuged at 1600 g for 10 minutes at 2°C. This washing process was repeated thrice. The resultant pellet was vigorously mixed with 0.1% Triton X-100 and was then recentrifuged at 8000 g for 30 minutes in a refrigerated centrifuge. The supernatant was used for biochemical assessments.

Semen samples were obtained from participants before and after treatment with two capsules of zinc sulfate (220 mg) per day for three months. The collected samples were categorized into three groups: group 1 (G1): healthy fertile subjects; group 2 (G2): patients with subfertility before treatment; and group 3 (G3): patients after treatment.

### Preparation of NTPC medium

The medium contained Tris buffer (20 mM, 0.242 g/100 ml), D-glucose (1.5 mM, 0.0027 g/100 ml), Ethylenediaminetetraacetic acid (EDTA) (0.4 mM, 0.0148 g/100 ml), NaCl (113 mM, 0.66 g/100 ml), Na2HPO4 (2.5 mM, 0.0355 g/100 ml), Na_2_HPO_4_ (2.5 mM, 0.3 g/100 ml), and CaCl_2_ (1.7 mM, 0.0188 g/100 ml). Finally, pH was adjusted to 7.4 using 0.1 M HCl. D-glucose was procured from Sigma, USA. Other chemicals were obtained from BDH Chemicals Ltd, Poole, Dorset, UK.

### Reagents and solutions

All reagents and solutions were obtained from standard commercial suppliers, were of analytical grade and were used without further purification.

### Biochemical methods

#### Determination of the adenosine deaminase activity

ADA was measured using the protocol described by Martinek (17), in which the ammonia produced by deamination reaction reacts with hypochlorite to form an intermediate, monochloramine, which in turn reacts with added phenol to form blue-color indophenols that have maximum absorption at 640 nm. The reaction mixture was composed of 0.5 ml of buffered substrate (adenosine; pH=7.05) and 0.05 ml of specimen incubated for 3 minutes at 37°C in a water bath. Then, 2.5 ml of phenol reagent and subsequently, 2.5 ml of hypochlorite reagent were added. Absorption was read at 640 nm, against the reagent blank. Test units activity of ADA activity is obtained from the calibration curve.

#### Determination of xanthine oxidase activity

XO activity was determined using Hadwan et al. (18) method. This method is based on the reaction between H_2_O_2_ and thiamine to produce fluorescent thiochrome with excitation and emission wavelengths of 370 and 425 nm, respectively. Reaction mixture consisted of 30 μl of specimen, 0.3 mM xanthine and 50 mM 3-aminotriazole dissolved in 1000 μl of 50 mM sodium phosphate buffer (pH=7.4). XO activity was obtained from the standard curve plotted for concentration of hydrogen peroxide against fluorescence intensity.

#### Determination of 5'-nucleotidase activity

U activity was done using Hadwan et al. (19) method, in which phosphate is liberated by the reaction of molybdate in the acidic medium leading to formation of a complex of phosphomolybdate, which is in turn reduced to unstable molybdenum blue. A volume of 0.2 mL of a specimen was taken, and then 0.1 mL of 0.02 M MnSO4 and 1.5 mL of 40 mM (pH=7.5) barbitone buffer, were added. One unit of activity of 5›-NU is defined as the release of 1 μmol inorganic phosphate per minute. The level of UA in serum was enzymatically measured using the Biomeghrib® kit (Morocco).

#### Statistical analysis

SPSS 21 software (SPSS Inc., Chicago, IL, USA) was used for statistical analysis. Results are expressed as mean, standard deviation (SD), standard error (SE), and range. Data were analyzed using one-way analysis of variance (ANOVA). The Kolmogorov Smirnov test was used to verify if data followed normal distribution. A significance level of P≤0.05 was considered to estimate differences in mean values of the following three groups: G1 (healthy donors), G2 (patients before treatment), and G3 (patients after treatment).

## Results

Table 1 presents the baseline values of the semen parameters. The ejaculated seminal fluids after treatment were reported in Table 1 in order to easily compare it with the baseline data. The patients in the present study were classified into three groups: G1 (healthy donors), G2 (patients before treatment), and G3 (patients after treatment). Seminal parameters were significantly decreased in the subfertile group (G2) compared to healthy donor group (G1). Seminal parameters were included normal sperm count (P=0.023), progressive sperm motility (P=0.001), and semen volume (P=0.042). The results presented significant improvements in ejaculate properties in the group treated with zinc compared to the same group before treatment with zinc. Seminal parameters were included the normal sperm count (P=0.03), progressive sperm motility (P=0.005), and semen volume (P=0.037).

The UA levels and 5’-NU, ADA, and XO activities of seminal plasma and spermatozoa in the patients and healthy groups are presented in Tables 2, 3, 4, and 5, respectively. As compared with healthy subjects (G1), the spermatozoa and seminal plasma of patients with asthenozoospermia (G2) indicated decreases in UA levels and 5’-NU activity but increases in ADA and XO activities. However, zinc supplementation restored the seminal plasma concentration of UA (P=0.034) and activities of 5’-NU (P=0.046), ADA (P=0.05) and XO (P=0.015) to normal levels in treated patients (G3).

**Table 1:** Parameters of ejaculated seminal fluids in asthenozoospermic patients and healthy subjects


Name of group	Volume (mL)	Sperm count (×10^6^)	Progressive sperm motility (%)	Normal sperm form (%)

Healthy donors (G1)	2.8 ± 0.53	77 ± 9	69 ± 8	38 ± 9
Patients before treatment (G2)	1.83 ± 0.66^*^(P=0.042)	47 ± 21^*^(P=0.023)	21 ± 9^*^(P=0.00)	21 ± 11(P=0.005)
Patients after treatment (G3)	2.39 ± 0.9^*^^*^(P=0.037)	70 ± 15(P=0.03)	39 ± 14^*^^*^(P=0.05)	33 ± 7^*^^*^(P=0.041)


Data are presented as mean ± SD. *; Significance versus group I (healthy donors) and **; Significance versus group II (patients before treatment).

**Table 2:** Uric acid levels in seminal plasma (µM/L) and spermatozoa (µM/108 spermatozoa) of asthenospermic patients and healthy subjects


Name of group	Source	Mean ± SD	± SE	95% confidence interval for mean		Compared groups		P value
				Lower bound	Upper bound			

G1	Seminal plasma	143.90 ± 44.44	5.73	124.68	163.11	1	2	0.034^*^
							3	0.185
G2	Seminal plasma	109.85 ± 53.48	6.9	86.72	132.98	2	1	0.034^*^
							3	0.001^*^
G3	Seminal plasma	164.98 ± 60.84	7.85	138.66	191.29	3	1	0.185
							2	0.001^*^
G1	Spermatozoa	77.61 ± 22.47	2.9	59.33	95.89	1	2	0.05^*^
							3	0.952
G2	Spermatozoa	53.65 ± 14.27	1.84	43.58	63.71	2	1	0.05^*^
							3	0.05^*^
G3	Spermatozoa	76.87 ± 24.17	3.12	53.45	100.3	3	1	0.952
							2	0.05^*^


*; Significance versus group I (healthy donors), SD; Standard deviation, and SE; Standard error.

**Table 3:** 5’-nucleotidase activity in seminal plasma (U/l) and spermatozoa (U/108 spermatozoa) of asthenospermic patients and healthy subjects


Name of group	Source	Mean ± SD	± SE	95% confidence Interval for mean		Compared groups		P value
				Lower bound	Upper bound			

G1	Seminal plasma	9.57 ± 2.68	0.34	6.71	12.43	1	2	0.046^*^
							3	0.062
G2	Seminal plasma	5.85 ± 1.86	0.24	3.18	8.52	2	1	0.046^*^
							3	0.000^*^
G3	Seminal plasma	13.04 ± 3.60	0.46	10.49	15.59	3	1	0.062
							2	0.000^*^
G1	Spermatozoa	17.09 ± 6.88	0.89	15.38	21.80	1	2	0.045^*^
							3	0.943
G2	Spermatozoa	11.30 ± 5.12	0.66	9.81	15.78	2	1	0.045^*^
							3	0.050^*^
G3	Spermatozoa	16.89 ± 5.96	0.76	13.89	19.98	3	1	0.943
							2	0.050^*^


*; Significance versus group I (healthy donors), SD; Standard deviation, and SE; Standard error

**Table 4:** Adenosine deaminase activity in seminal plasma (U/l) of asthenospermic patients and healthy subjects


Name of group	Mean ± SD	± SE	95% confidence interval for mean		Compared groups		P value
			Lower bound	Upper bound			

G1	22.39 ± 4.87	0.62	15.68	29.10	1	2	0.050^*^
						3	0.476
G2	41.18 ± 12. 42	1.06	29.29	53.08	2	1	0.050^*^
						3	0.016^*^
G3	15.92 ± 5.01	0.64	19.93	29.90	3	1	0.476
						2	0.016^*^


*; Significance versus group I (healthy donors), SD; Standard deviation, and SE; Standard error

**Table 5:** Xanthine oxidase activity in seminal plasma (mU/l) and spermatozoa (mU/108 spermatozoa) of asthenospermic patients and healthy subjects


Name of group	Source	Mean ± SD	± SE	95% confidence interval for mean		Compared groups		P value
				Lower bound	Upper bound			

G1	Seminal plasma	128 ± 34.10	4.04	98.06	188.28	1	2	0.015^*^
							3	0.555
G2	Seminal plasma	218 ± 53.11	6.85	173.88	263.64	2	1	0.015^*^
							3	0.050^*^
G3	Seminal plasma	151 ± 46.78	6.03	97.09	201.02	3	1	0.555
							2	0.050^*^
G1	Spermatozoa	110.65 ± 38.27	4.94	77.46	143.83	1	2	0.035^*^
							3	0.436
G2	Spermatozoa	199.88 ± 57.97	7.48	151.40	248.34	2	1	0.035^*^
							3	0.050^*^
G3	Spermatozoa	143.94 ± 63.40	8.18	97.18	205.07	3	1	0.436
							2	0.050^*^


*; Significance versus group I (healthy donors), SD; Standard deviation, and SE; Standard error

## Discussion

The results of the current study showed that supplementation of zinc enhanced the semen quality in infertile men. Our findings verified the data reported by a previous study (6) which linked the enhancement of semen quality to the biological properties of zinc, such as spermatogenesis induction, stimulation of sex organs growth , activation of 5α-reductase enzyme that is necessary for the conversion of testosterone into the chemically active form, 5α-dihydrotestosterone and increment of the activity of Zn-containing enzymes that play significant roles in sperm motility such as lactate dehydrogenase and sorbitol dehydrogenase. Fallah et al. (3), highlighted the importance of Zn content of seminal plasma for men’s health, normal sperm function, fertilization and germination. On the other hand, highly toxic levels of zinc may have harmful effects on sperm quality.

Compared with the control group, UA concentrations were significantly decreased in the spermatozoa and seminal plasma of the patients with asthenozoospermia. Former studies did not report the main reason underlying decrement of UA levels in patients with asthenozoospermia (20). However, decreased UA could be related to elevated peroxynitrite levels in the seminal plasma of patients with asthenozoospermia. UA acts as a scavenger of peroxynitrate, to produce nitrated UA (21).

Oxidative stress might be the main reason for the depletion of UA in the spermatozoa and seminal plasma of patients with asthenozoospermia; UA was speculated to have ROS scavenging activity, and regular treatment with UA was documented to enhance antioxidant capacity (22). Mikami et al. (23) demonstrated a significant reverse correlation between oxidative stress and UA levels. UA reacts with ROS and convert them to an oxidized form, in mammalian tissues. Its action as an antioxidant is a so-called comprehensive mechanism in mammalian tissues, where it may offer an oxidizable cosubstrate role to any attacking ROS, therefore, protecting the macromolecules from oxidative stress injury (20). In addition, UA preserves the integrity of cell membranes by preventing membrane LPO. It also participates in the stabilization of vitamin C antioxidant activity in the seminal plasma (24).

Administration of zinc salt supplements increases the UA concentration in asthenozoospermic seminal plasma to the reference range; this may be attributed to two mechanisms. First, it improves the total antioxidant status (6). Second, they induce the production of metallothioneins, which are low molecular mass zincbinding proteins (5) that remove peroxynitrite from seminal plasma.

Hydrolysis of ATP produces adenosine that adapts to various reproductive functions, such as those involving contraction, steroidogenesis, and maintenance of fluid composition. Interestingly, adenosine might act as a key capacitative modulator for mammalian spermatozoa to achieve fertilization (25). Extracellular nucleotide levels are influenced by cell surface ectonucleotidases. 5’-NT (EC 3.1.3.5) is a glycoprotein tightly bound to the membrane of mammalian spermatozoa and is an ectoenzyme with its active site facing the external medium (26). The 5’-NT of seminal plasma is a metalloprotein containing two zinc atoms per subunit of dimeric protein. Removal of the two zinc atoms from the enzyme molecule, results in a completely inactive apoenzyme (27). This enzyme is generally used in diagnosing plasma membrane abnormalities. The development of sperm fertilization and migration primarily depends upon the plasma membrane. A decrease in 5’-NT activity is usually regarded as a damage to the membrane architecture caused by elevated ROS concentrations in biological samples (28). Extracellular AMP is hydrolyzed by 5’- NT to free phosphate and adenosine. The present study demonstrates significant depletion of 5’-NT activity in the semen of patients with asthenozoospermia. This decrease may cause spermatozoal damage owing to exposure to ROS, which may consequently disturb membrane integrity and function. The most commonly proposed reason for this depletion has been the oxidative modification of 5’-NT sulfhydryl (SH) groups and the reaction with LPO end-product. This impression comes from a previous study which reported powerful inhibition of 5’-NT activity by damaged sulfhydryl groups’ compared with several other enzymes (29). Also, decreased 5’-NT activity may be caused by elevated NO levels in the asthenozoospermic semen. Siegfried et al. (30) reported that NO interacts with ecto- 5’-NT and S-nitrosylation of 5’-NT probably results in inhibition of the enzyme activity. Overproduction of NO may cause an impairment of 5’-NT activity *in vivo*.

The enzyme that is subsequently produced in nucleotide catabolism is ADA, (EC. 3.5.4.4), also known as adenosine aminohydrolase. This enzyme irreversibly deaminates 2’-deoxyadenosine and adenosine to deoxyinosine and inosine. ADA is widely distributed among prokaryotic and eukaryotic cells. It is essential for the proliferation, maturation, and differentiation of lymphocytes (31). The active site of this metalloenzyme consists of Zn^2+^ that is present in the deep site of cavity and coordinates with enzyme substrate and four amino acid residues (His 15, 17, and 214 and Asp 295). Zn^2+^ is considered a sole cofactor of ADA activity (32).

The ADA values in the present study were found to be significantly elevated in asthenozoospermic patients compared with controls. The proposed mechanisms for increased ADA activity could be related to the increase of leukocyte levels and inflammatory conditions in seminal plasma in patients with asthenozoospermia (2). A previous study also reported that ADA activity increased in inflammatory diseases, indicating activation and proliferation of T-cells. Thus, ADA is regarded as a T-cell activation marker (33). Erkiliç et al. (34) documented that ADA increases the overproduction of ROS, such as H2O2, O_2_^-^, NO, and 1O2. The overproduction of ROS generates oxidative stress, which amplifies inflammatory responses by propagating LPO adjacent to the membrane; this may initiate the development of spermatozoal dysfunction.

Decreased 5’-NT activity and increased ADA activity inevitably lead to decreased adenosine levels. These conditions lead to increased oxidative stress and generate unwanted complications because the amount of remaining adenosine is insufficient to perform its physiological functions. To exhaust extracellular adenosine, adenosine receptors play a role in the lowering vascular tone (35). In addition, adenosine is an essential anti-inflammatory agent, which suppresses tumor necrosis factor-alpha (TNF-α) production in monocytes and macrophages, thereby inhibiting the liberation of arachidonic acid and leukotriene production in neutrophils (36). Adenosine acts as an endogenous activator in antioxidant enzyme pathways (37). The 5’NU/ADA dynamic ratio was found to be increased in the group treated with zinc, indicating that adenine nucleotide metabolism may tend to stimulate adenosine production and therefore increase the pool of adenosine. The zinc supplementation decreased ADA activity owing to its anti-apoptotic and antioxidant properties.

The doses used in the present study were the same as those used in previous studies. Zinc sulfate (ZnSO_4_) was used as an antioxidant in previous clinical studies (38). The dosage of ZnSO_4_ used in previous clinical trials ranged from 66 to 500 mg, and the treatment duration ranged from 13 to 26 weeks. The results of these clinical trials have indicated positive benefits of ZnSO_4_. No negative results for use of ZnSO_4_ were reported in the previous studies; hence, investigators inferred that the dosage used was safe.

XO is a metalloenzyme containing iron, sulfur, and molybdenum in its active site; it has various functions and is widely distributed in the endothelial cells of sinusoids and capillaries (12). XO has two forms and functions: xanthine dehydrogenase (more predominant) and XO. The predominant form of XO oxidizes hypoxanthine to xanthine and UA via its dehydrogenase activity and generates NADH, whereas the minor XO with oxidase activity, produces O_2_^-^. The predominant XO could be modified to oxidase XO either by reversible oxidation of thiol groups in its active site or by an irreversible proteolytic attack. The formation of O_2_^-^ by XO was intensively studied in experimental oxidative stress in seminal fluids (39).

The results of the present study showed a significant elevation in XO activity in asthenozoospermic samples compared with the control group. Increased XO activity could be related to the conversion of the dehydrogenase form of XO into the oxidase form, by reversible oxidation of thiol groups or by irreversible proteolytic attack caused by elevated peroxynitrite levels. Also, increased activity of ADA that elevates the xanthine pool could contribute to increased XO activity. Thus, the high activity of XO can be explained by the high xanthine levels present in the semen of patients with asthenozoospermia, because xanthine is one of the substrates of XO. Therefore, the elevated levels of xanthine require elevated XO activity, which might tend to generate high oxidative stress.

An increase in the activity of the ADA and XO enzymes with contradictory low levels of UA in patients group, is attributed to the paradoxical effect of elevated oxidative stress on ADA enzyme, XO enzyme and UA.

Supplementing Zn as an inhibitor of XO activity has been paid great attention. Zinc supplementation restores XO in the seminal plasma and spermatozoa of patients with asthenozoospermia to reference values. You et al. (40) documented that Zn compounds act as XO inhibitors. XO is a rate-limiting enzyme in the degradation pathway of purine nucleotide. Because XO is considered one of the major generators of ROS, decreases in its activity may contribute to the reduction of LPO levels by dietary zinc supplementation. A limitation of the present trial was that the measurements of enzymes were completed without blinding the biochemist to the investigational groups, which has the potential for bias. Also, serum zinc status of subjects was not determined, and the study lacked a placebo-treated group. However, potential bias was reduced by random assignment of participants and through following the standardized protocol by the investigator. Although, zinc sulfate was previously used several times to treat asthenozoospermia, the present study should be repeated in different target populations to establish the external validity.

## Conclusion

Zinc supplementation restores UA levels and the activities of enzymes involved in the urate pathway (XO and ADA), in the seminal plasma and spermatozoa of patients with asthenozoospermia, to reference values. Supplementation of Zn compounds enhances the qualitative and quantitative properties of semen.

## References

[1] Dubey CK, Sharma A, Khinchi MP (2017). A Review on common factors affecting a semen quality of male. Asian Journal of Pharmaceutical Research and Development.

[2] Alahmar AT (2018). The effects of oral antioxidants on the semen of men with idiopathic oligoasthenoteratozoospermia. Clin Exp Reprod Med.

[3] Fallah A, Mohammad-Hasani A, Colagar AH (2018). Zinc is an essential element for male fertility: a review of Zn roles in men’s health, germination, sperm quality, and fertilization. J Reprod Infertil.

[4] Hadwan MH, Almashhedy LA, Alsalman AR (2012). Oral zinc supplementation restore high molecular weight seminal zinc binding protein to normal value in Iraqi infertile men. BMC Urol.

[5] Narasimhaiah M, Arunachalam A, Sellappan S, Mayasula VK, Guvvala PR, Ghosh SK (2018). Organic zinc and copper supplementation on antioxidant protective mechanism and their correlation with sperm functional characteristics in goats. Reprod Domest Anim.

[6] Hadwan MH, Almashhedy LA, Alsalman ARS (2013). The key role of zinc in enhancement of total antioxidant levels in spermatozoa of patients with asthenozoospermia. American Journal of Molecular and Cellular Biology.

[7] Baker MA, Netherton J, Aitken RJ, Henkel R, Samanta L, Agarwal A (2019). From past to present: an historical overview of the concept of spermatozoa, reactive oxygen species, and male-factor infertility. Oxidants, antioxidants and impact of the oxidative status in male reproduction.

[8] de Andrade AFC, Arruda RP, Torres MA, Pieri NCG, Leite TG, Celeghini ECC (2018). Nitric oxide in frozen-thawed equine sperm: Effects on motility, membrane integrity and sperm capacitation. Anim Reprod Sci.

[9] Saleem K, Begum TN, Ilyas MM (2017). Effect of Azima Tetracantha on mitochondrial membrane bound enzymes in liver on carbon tetrachloride induced oxidative stress in rats. Int J Sci Res.

[10] Xie D, Lu C, Zhu Y, Zhu S, Yang EJ, Jin X (2018). Analysis on the association between sperm DNA fragmentation index and conventional semen parameters, blood microelements and seminal plasma ROS in male patients with infertility. Exp Ther Med.

[11] Aitken RJ, De Iuliis GN, Drevet JR (2019). Role of oxidative stress in the etiology of male infertility and the potential therapeutic value of antioxidants.Oxidants, antioxidants and impact of the oxidative status in male reproduction.Academic Press.

[12] Banihani SA (2018). Role of uric acid in semen. Biomolecules.

[13] Kimoloi S (2018). Modulation of the de novo purine nucleotide pathway as a therapeutic strategy in mitochondrial myopathy. Pharmacol Res.

[14] Borea PA, Gessi S, Merighi S, Vincenzi F, Varani K (2018). Pharmacology of adenosine receptors: the state of the art. Physiol Rev.

[15] Kadam P, Bhalerao S (2010). Sample size calculation. Int J Ayurveda Res.

[16] Lu JC, Huang YF, Lü NQ (2010). WHO laboratory manual for the examination and processing of human semen: its applicability to andrology laboratories in China. Zhonghua Nan Ke Xue.

[17] Martinek RG (1963). Micromethod for estimation of serum adenosine deaminase. Clin Chem.

[18] Hadwan MH, Almashhedy LA, Alsalman AS (2014). Seminal xanthine oxidase, Appropriate fluorometric assay for the examination of spermatozoa disorders. Biotechnol, an Indian journal.

[19] Hadwan M, Almashhedy LAM, Alsalman AR (2012). New optimized method for assaying 5’-Nucletidase in biological fluids. Int J Biotech Biochem.

[20] Das P, Chaudhari AR, Dhawan A, Singh R (2009). Possible role of uric acid as an effective antioxidant in human seminal plasma. Biom Res.

[21] Skinner KA, White CR, Patel R, Tan S, Barnes S, Kirk M (1998). Nitrosation of uric acid by peroxynitrite formation of a vasoactive nitric oxide donor. J Biol Chem.

[22] Cheng S, Yang Y, Zhou Y, Xiang W, Yao H, Ma L (2018). Influence of different concentrations of uric acid on oxidative stress in steatosis hepatocytes. Exp Ther Med.

[23] Mikami T, Yoshino Y, Ito A (2000). Does a relationship exist between the urate pool in the body and lipid peroxidation during exercise?. Free Radic Res.

[24] Alta’ee AH, Majeed HA, Hadwan MH (2018). The levels of vitamin E in seminal fluids and their association with male infertility: Short review. Research Journal of Pharmacy and Technology.

[25] Pu F, Ren J, Qu X (2018). Nucleobases, nucleosides, and nucleotides: versatile biomolecules for generating functional nanomaterials. Chem Soc Rev.

[26] Alm‐Kristiansen AH, Standerholen FB, Bai G, Waterhouse KE, Kommisrud E (2018). Relationship between post‐thaw adenosine triphosphate content, motility and viability in cryopreserved bovine semen applying two different preservation methods. Reprod Domest Anim.

[27] Dar MR, Singh M, Sharma R, Thakur S, Sheikh AA, Bhat SA (2018). Bovine fertility as regulated by sperm binding proteins: a review. Asian J Anim Vet Adv.

[28] Viviani LG, Piccirillo E, Cheffer A, de Rezende L, Ulrich H, Carmona- Ribeiro AM (2018). Be aware of aggregators in the search for potential human ecto-5′-nucleotidase inhibitors. Molecules.

[29] Al-Haj L, Khabar KSA (2018). The intracellular pyrimidine 5′-nucleotidase NT5C3A is a negative epigenetic factor in interferon and cytokine signaling. Sci Signal.

[30] Siegfried G, Amiel C, Friedlander G (1996). Inhibition of ecto-5’-nucleotidase by nitric oxide donors.Implications in renalepithelial cells. J Biol Chem.

[31] Pirinççi N, Kaya TY, Kaba M, Ozan T, Geçit İ, Özveren H, Eren H, Ceylan K (2017). Serum adenosine deaminase, catalase, and carbonic anhydrase activities in patients with renal cell carcinoma. Redox Rep.

[32] Moreno E, Canet J, Gracia E, Lluís C, Mallol J, Canela EI (2018). Molecular evidence of adenosine deaminase linking adenosine A2A receptor and CD26 proteins. Front Pharmacol.

[33] Zuikov SA, Borzenko BG, Shatova OP, Bakurova EM, Polunin GE (2014). Correlation of nucleotides and c arbohydrates metabolism with pro-oxidant and antioxidant systems of erythrocytes depending on age in patients with colorectal cancer. Exp Oncol.

[34] Erkiliç K, Evereklioglu C, Çekmen M, Özkiris A, Duygulu F, Dogan H (2003). Adenosine deaminase enzyme activity is increased and negatively correlates with catalase, superoxide dismutase and glutathione peroxidase in patients with Behçet’s disease: original contributions/clinical and laboratory investigations. Mediators Inflamm.

[35] Kazemi MH, Raoofi Mohseni S, Hojjat‐Farsangi M, Anvari E, Ghalamfarsa G, Mohammadi H, Jadidi‐Niaragh F (2018). Adenosine and adenosine receptors in the immunopathogenesis and treatment of cancer. J Cell Physiol.

[36] Flamand N, Boudreault S, Picard S, Austin M, Surette ME, Plante H (2000). Adenosine, a potent natural suppressor of arachidonic acid release and leukotriene biosynthesis in human neutrophils. Am J Respir Crit Care Med.

[37] Gabazza EC, Hayashi T, Ido M, Adachi Y, Suzuki K (2002). Adenosine inhibits thrombin-induced expression of tissue factor on endothelial cells by a nitric oxide-mediated mechanism. Clin Sci (Lond).

[38] Hadwan MH, Almashhedy LA, Alsalman AR (2015). Oral zinc supplementation restores superoxide radical scavengers to normal levels in spermatozoa of Iraqi asthenospermic patients. Int J Vitam Nutr Res.

[39] Saez F, Hong NJ, Garvin JL (2018). NADPH oxidase 4-derived superoxide mediates flow-stimulated NKCC2 activity in thick ascending limbs. Am J Physiol Renal Physiol.

[40] You ZL, Shi DH, Zhu HL (2006). The inhibition of xanthine oxidase by the Schiff base zinc (II) complex. Inorg Chem Commun.

